# Potential Usefulness of* Streptococcus pneumoniae* Extracellular Membrane Vesicles as Antibacterial Vaccines

**DOI:** 10.1155/2017/7931982

**Published:** 2017-01-22

**Authors:** Chi-Won Choi, Edmond Changkyun Park, Sung Ho Yun, Sang-Yeop Lee, Seung Il Kim, Gun-Hwa Kim

**Affiliations:** ^1^Division of Bioconvergence Analysis, Korea Basic Science Institute, Daejeon, Republic of Korea; ^2^Tunneling Nanotube Research Center, Division of Life Science, Korea University, Seoul 02841, Republic of Korea; ^3^Department of Bio-Analytical Science, University of Science and Technology, Daejeon, Republic of Korea; ^4^Center for Convergent Research of Emerging Virus Infection, Korea Research Institute of Chemical Technology, Daejeon, Republic of Korea; ^5^Department of Functional Genomics, University of Science and Technology, Daejeon, Republic of Korea

## Abstract

The secretion of extracellular membrane vesicles (EMVs) is a common phenomenon that occurs in archaea, bacteria, and mammalian cells. The EMVs of bacteria play important roles in their virulence, biogenesis mechanisms, and host cell interactions. Bacterial EMVs have recently become the focus of attention because of their potential as highly effective vaccines that cause few side effects. Here, we isolated the EMVs of* Streptococcus pneumoniae* and examined their potential as new vaccine candidates. Although the* S. pneumoniae* bacteria were highly pathogenic in a mouse model, the EMVs purified from these bacteria showed low pathological activity both in cell culture and in mice. When mice were injected intraperitoneally with* S. pneumoniae* EMVs and then challenged, they were protected from both the homologous strain and another pathogenic serotype of* S. pneumoniae*. We also identified a number of proteins that may have immunogenic activity and may be responsible for the immune responses by the hosts. These results suggest that* S. pneumoniae* EMVs or their individual immunogenic antigens may be useful as new vaccine agents.

## 1. Introduction

*Streptococcus pneumonia* is an alpha-hemolytic Gram-positive encapsulated aerobic diplococcus bacterium that is the main causative pathogen of community-acquired respiratory tract infections.* S. pneumoniae* is generally considered to be a human pathogen because it causes a number of human diseases, including otitis, sinusitis, bacterial meningitis, sepsis, and pneumonia [1]. The people who are most affected by this organism are children and individuals with immature or compromised immune systems, such as patients with diabetes or acquired immunodeficiency syndrome [2, 3]. Moreover,* S. pneumoniae* has been isolated from various animals, including guinea pigs, cats, horses, dogs, and gorillas. These animals all exhibit* S. pneumoniae-*related clinical symptoms [4]. These* S. pneumonia*-infected animal hosts thus may serve as an extrahuman reservoir from which the pathogen can be transmitted to humans. Therefore, to prevent* S. pneumoniae* infections, it is necessary to vaccinate both humans and animals such as pets. Pneumococcal conjugate vaccines are widely used because they are highly effective in preventing pneumococcal invasive disease. A recent review reported that failure of pneumococcal conjugate vaccines is rare, but irrespective of vaccine or schedule [5]. This may lead us to evaluate EMVs of* S. pneumoniae* for vaccine candidates.

Bacterial extracellular membrane vesicles (EMVs) are spherical vesicles that are secreted by a variety of bacteria. These vesicles measure 20–250 nm in diameter and contain various biologically active proteins that are required for bacterial nutrient acquisition, biofilm formation, and pathogenesis [6]. Since bacterial EMVs are nonviable and yet induce a host immune response, they have great potential as acellular antibacterial vaccines. In particular, the EMV surface proteins can act as antigens that induce adaptive immune responses in the host.

Here, we isolated nonpathogenic (noncapsular)* S. pneumoniae* EMVs and examined their ability to serve as next-generation vaccines that protect against infections with pathogenic or nonpathogenic bacteria. We also identified the antigenic protein components of the EMVs.

## 2. Materials and Methods

### 2.1. Bacterial Strains and Growth Conditions

*Streptococcus pneumoniae* BAA-255 was purchased from the American Type Culture Collection (ATCC, http://www.atcc.org, Manassas, VA, USA).* Streptococcus pneumoniae* KCCM-41569 was obtained from the Korean Culture Center of Microorganisms (KCCM, http://www.kccm.or.kr). The bacteria were grown to an OD_600_ of approximately 1.0 in a 5% CO_2_ atmosphere at 37°C in Todd-Hewitt broth supplemented with 0.5% yeast extract and bacteria cells were counted by using Quantum Tx microbial cell counter (Logos Biosystems, Korea).

### 2.2. Purification of EMVs

EMVs of* S. pneumoniae *BAA-255 were purified from bacterial culture supernatants by using the method of Choi et al. [7], with some modifications. Briefly, the bacterial cells were removed from the bacterial culture by centrifugation at 16,000 ×g for 20 min. The supernatants were then filtered through a 0.2 *μ*m hollow fiber membrane (GE Healthcare, Little Chalfont, Buckinghamshire, UK) to further remove residual cells and debris. The EMVs were concentrated and ultrafiltrated by using a QuixStand Benchtop System fitted with a 500 kDa hollow fiber membrane (GE Healthcare). The resulting EMVs were precipitated by ultracentrifugation at 150,000 ×g for 3 h at 4°C and the EMV-containing pellets were suspended in 1.0–2.0 mL of phosphate-buffered saline (PBS). Finally, the EMV solution was layered over a sucrose gradient (2.5, 1.6, and 0.6 M sucrose) to remove contaminating proteins. Each fraction was centrifuged at 200,000 ×g for 20 h at 4°C and the sucrose was removed by ultracentrifugation at 150,000 ×g for 3 h at 4°C. The protein concentration was determined by using a bicinchoninic acid assay (Thermo Scientific, Waltham, MA, USA). The purified EMVs were stored at −80°C until required.

### 2.3. Transmission Electron Microscope (TEM) Observation of EMVs

For TEM analysis, 5 *μ*L of the 5 mg/mL purified EMV sample was loaded onto a freshly glow-discharged holey carbon EM grid (Quantifoil R 2/2, Quantifoil Micro Tools GmbH, Germany). Semiautomated sample verification was performed by using a Vitrobot Mark IV (FEI Company, Eindhoven, Netherlands) at 4°C and 90–100% relative humidity. The vitrified sample was imaged under low dose conditions by using a Tecnai G^2^ Spirit TEM (FEI Company, Eindhoven, Netherlands) that was operated at 120 kV acceleration voltage. Images were recorded by using an UltraScan 4000 charge-coupled device camera (Gatan Inc., Pleasanton, CA, USA) at a nominal magnification of ×26,000 and −1–2 *μ*m underfocus.

### 2.4. Viability and Apoptosis of Human Lung Cancer Cells after Treatment with Bacteria and EMVs

Briefly, the human lung epithelial adenocarcinoma line A549 was cultured in 96-well plates (1 × 10^5^ cells/well) with* S. pneumoniae* bacteria or EMVs for 24 h, after which cell viability and apoptosis were measured as described previously [7]. The culture medium was RPMI 1640 (Gibco, Waltham, MA, USA) supplemented with heat-inactivated 10% FBS and antibiotics (Gibco). The ranges of multiplicity of infection (MOI) of* S. pneumoniae* that were used to infect the A549 cells were 0.001, 0.1, 10, and 1,000 for* S. pneumoniae* BAA-255 and 0.001, 0.01, 0.1, and 1 for* S. pneumoniae* KCCM-41569. The concentrations of* S. pneumoniae* BAA-255 EMVs were 50, 100, and 200 *μ*g protein in 100 *μ*L of culture medium. To measure cell viability, the cells were stained with acridine orange and DAPI (ChemoMetec, Allerød, Denmark). To measure apoptosis, the cells were stained with FITC-conjugated Annexin V, propidium iodide, and Hoechst (ChemoMetec) according to the manufacturer's instructions. The stained cells were then analyzed in a NucleoCounter NC-3000 image cytometer (ChemoMetec).

### 2.5. Vaccination of Mice Followed by Bacterial Challenge

Six-week-old female C57BL6/J mice were purchased from DBL (Korea) and housed under specific pathogen-free conditions. At the age of 8 weeks, the mice received an intraperitoneal injection (200 *μ*g in PBS) of* S. pneumoniae* BAA-255 EMVs. This was repeated twice at intervals of 2 weeks. Two weeks after the third immunization, the mice were infected intraperitoneally with a lethal dose of nonpathogenic* S. pneumoniae* BAA-255 (1 × 10^8^ cfu) or pathogenic* S. pneumoniae* KCCM-41569 (1 × 10^3^ cfu). Survival was monitored for 7 days. Control mice were immunized with equivalent volumes of PBS and then challenged. All animal experiments were reviewed and approved by the Animal Ethics Committee at the Korea Basic Science Institute (approval number KBSI-AEC 1314).

### 2.6. Identification of the Proteins in EMVs

The proteins in* S. pneumoniae *EMVs were identified by using one-dimensional electrophoresis-liquid chromatography tandem mass spectrometry (1-DE-LC-MS/MS) as described previously [7]. In brief, purified EMVs were lysed and the protein lysates were separated by 12% sodium dodecyl sulfate-polyacrylamide gel electrophoresis (SDS-PAGE), followed by tryptic in-gel digestion. The digested peptide fractions were then loaded onto a 10 cm × 75 *μ*m inner diameter C18 reverse-phase column (PROXEON, Odense, Denmark; Aqua; particle size, 5 *μ*m) and subjected to a flow rate of 120 nL/min. The peptides were eluted with a gradient of 0–80% acetonitrile containing 0.1% formic acid for 80 min. All MS and MS/MS spectra were acquired by using a Thermo Finnigan LTQ mass spectrometer (San Jose, CA, USA). Each full MS (*m*/*z* range of 400–2,000) scan was followed by three MS/MS scans of the most abundant precursor ions in the MS spectrum, with dynamic exclusion enabled. Proteins were identified by using MASCOT software (ver. 2.4; Matrix Science Inc., USA). A* S. pneumoniae* R6 protein database (https://www.ncbi.nlm.nih.gov/) was used to analyze the MS/MS data. Carbamidomethylation of cysteine (+57 Da), oxidation of methionine (+16 Da), and propionamide of cysteine (+71 Da) were considered to be variable protein modifications. The exponentially modified protein abundance index (emPAI) was generated by using MASCOT software and the mol% was calculated according to emPAI values. Each sample underwent the MS/MS analysis three times.

## 3. Results

### 3.1. Production of EMVs from* S. pneumoniae*

**Figure 1:**
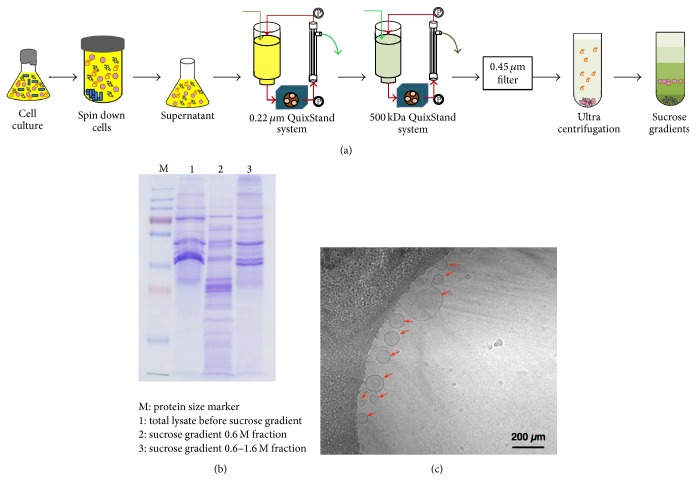
Purification of* Streptococcus pneumoniae* BAA-255 extracellular membrane vesicles (EMVs). (a) Summary of the method used to prepare* S. pneumoniae* BAA-255 EMVs. (b) SDS-PAGE of the EMVs before and after sucrose gradient fractionation. (c) Transmission electron microscopy of the EMVs. Red arrows indicate EMVs.

We first examined whether* S. pneumoniae* produced EMVs. Thus,* S. pneumoniae* BAA-255 was grown in Todd-Hewitt broth that was supplemented with 0.5% yeast extract until the late exponential phase (OD_600 _~ 1.0). The bacterial cells were precipitated by centrifugation and the supernatant was prepared. The supernatant was ultrafiltered to remove cells and cellular debris and sucrose gradient centrifugation was performed to remove contamination with other protein complexes (Figure 1(a)). The EMVs were enriched between 0.6 and 1.6 M sucrose (Figure 1(b)). TEM examination confirmed the presence of* S. pneumoniae* BAA-255 EMVs. The diameter of the EMVs ranged from 40 nm to 200 nm (Figure 1(c)). This is similar to the diameters of other bacterial EMVs.

### 3.2. Cytotoxic Effects of* S. pneumonia* EMVs

**Figure 2:**
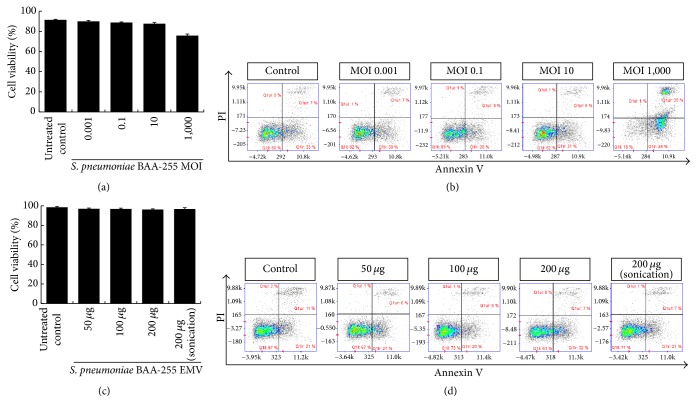
Cytotoxicity of intact* S. pneumoniae* BAA-255 and its EMVs. A549 cells were treated with various concentrations of intact* S. pneumoniae* BAA-255 and cell viability (a) and apoptosis (b) were analyzed. A549 cells treated with various concentrations of* S. pneumoniae* BAA-255 EMVs and cell viability (c) and apoptosis (d) were analyzed.

The endotoxic activity of virulence factors in EMVs significantly hampers their usefulness as vaccines. Therefore, we assessed whether nonpathogenic* S. pneumoniae* BAA-255 and its EMVs induce host cell damage by treating A549 cells with various concentrations of* S. pneumoniae* BAA-255 bacteria and EMVs. Cell viability and apoptosis assays showed that high concentrations (1 × 10^8^ cfu, MOI 1,000) of* S. pneumoniae* BAA-255 cells slightly reduced cell viability and induced apoptosis (Figures 2(a) and 2(b)). However, when the cells were treated with EMVs from* S. pneumoniae* BAA-255, cell viability and apoptosis rates were unchanged, even in the presence of high concentrations (200 *μ*g) of EMVs (Figures 2(c) and 2(d)). By contrast, a pathogenic strain of* S. pneumoniae,* namely, KCCM-41569, killed A549 cells even at very low concentrations (1 × 10^3^ cfu, MOI 0.01) (Figure S1) (see Supplementary Material available online at https://doi.org/10.1155/2017/7931982). These results suggest that EMVs from nonpathogenic* S. pneumoniae* BAA-255 are not cytotoxic.

### 3.3. Immunization with* S. pneumoniae* EMVs

**Figure 3:**
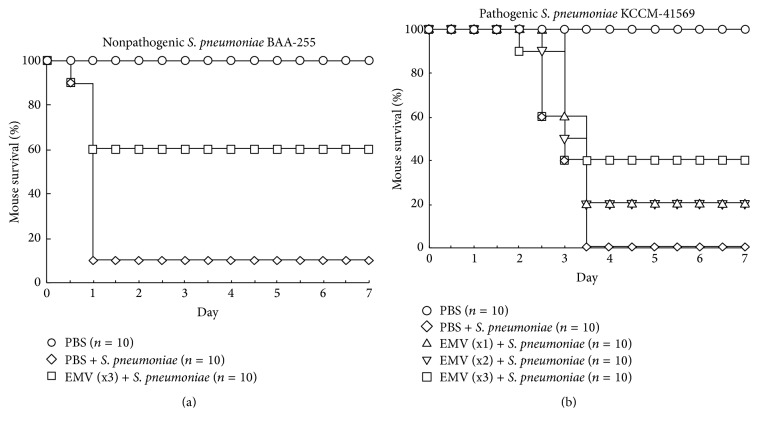
Survival of vaccinated mice after infection with* Streptococcus pneumoniae*. Mice (8 weeks old) were vaccinated intraperitoneally with* S. pneumoniae* BAA-255 extracellular membrane vesicles. After intraperitoneal inoculation with 1 × 10^8^ cfu of nonpathogenic* S. pneumoniae* BAA-255 or 1 × 10^3^ cfu of pathogenic* S. pneumoniae* KCCM-41569, survival over 7 days was assessed. Equivalent volumes of phosphate-buffered saline served as a vaccine control.

To evaluate the potential and efficacy of EMVs as a new vaccine against* S. pneumoniae *infection, mice were vaccinated with* S. pneumoniae* BAA-255 EMVs and then challenged with live* S. pneumoniae *BAA-255 bacteria. After bacterial challenge, 10% of the control mice survived. By contrast, 60% of the EMV-vaccinated and challenged mice survived (Figure 3(a)). This suggests that vaccination with* S. pneumoniae* EMVs protects mice against* S. pneumoniae*. Control and EMV-vaccinated mice were also challenged with KCCM-41569, a pathogenic strain of* S. pneumoniae*. None of the control mice survived infection with 1 × 10^3^ cfu of* S. pneumoniae* KCCM-41569. However, some of the vaccinated mice did survive: 40% survived after three immunizations and 20% survived after a single or double immunization (Figure 3(b)). These results show that immunization with EMVs isolated from a nonpathogenic* S. pneumoniae* BAA-255 strain not only protected against homologous challenge but also provided cross-protection against challenge with a pathogenic heterologous strain.

### 3.4. Identification of* S. pneumoniae* EMV Proteins

**Figure 4:**
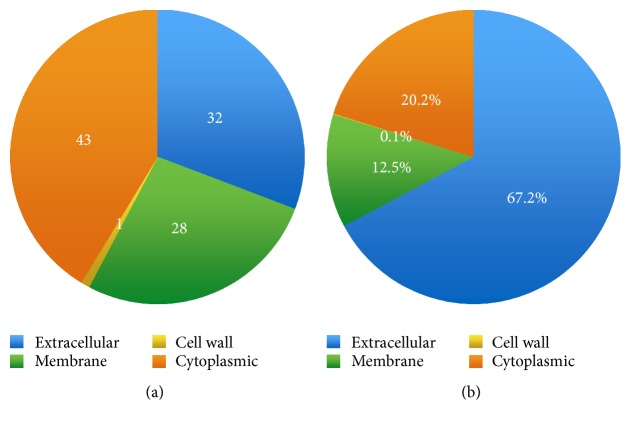
Subcellular localization of proteins identified in* Streptococcus pneumoniae* BAA-255 extracellular membrane vesicles (EMVs). The subcellular localization of the different proteins identified in the EMVs according to the number of proteins (a) or the total amount of protein (b) is shown. The subcellular localization of the proteins was determined by using CELLO (http://cello.life.nctu.edu.tw).

LC-based proteomic analysis was performed to identify the protein components of* S. pneumoniae* EMVs. 1D-LC-MS/MS analysis identified a total of 104 proteins in* S. pneumoniae* BAA-255 EMVs (Supplementary Table S1). A cell location prediction program was then used to determine the subcellular localization of the identified proteins. Of the 104 proteins identified in* S. pneumoniae* EMVs, 32 were extracellular, 28 were from the membrane, one was from the cell wall, and 43 were cytoplasmic (Figure 4(a)). Of the 104 identified proteins, the 32 extracellular proteins accounted for more than half (67.2%) of the total protein in* S. pneumoniae* EMVs (Figure 4(b)).

**Figure 5:**
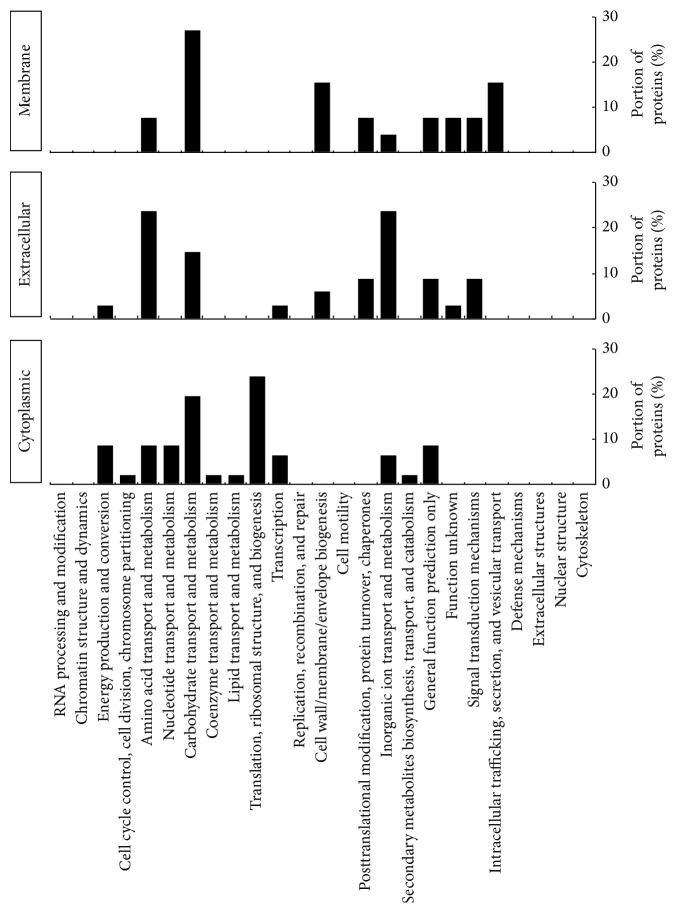
Functional annotation of the* Streptococcus pneumoniae* extracellular membrane vesicle proteins. The proteins were clustered according to their putative functions, which were determined by COGs (Clusters of Orthologous Groups).

The identified proteins were then analyzed by using a bioinformatics tool (Clusters of Orthologous Groups (COGs)) to determine the putative function of* S. pneumoniae* EMVs. The proteins in* S. pneumoniae* EMVs were mainly involved in the transport and metabolism of biomaterials such as amino acids and carbohydrates, as well as inorganic ions (Figure 5).

## 4. Discussion

Vaccines that prevent viral and bacterial diseases significantly improve public health. There are three types of vaccines against these microorganisms, namely, attenuated live vaccines, inactivated vaccines, and subunit vaccines. Although attenuated live and inactivated vaccines provide high levels of protection against viral and bacterial disease, there are concerns that they also elicit toxic side effects due to the presence of virulence factors. While this problem is overcome by subunit vaccines, they in turn are less effective and more expensive to produce than attenuated live or inactivated vaccines [8]. One way to sidestep these problems with existing antibacterial vaccine approaches is to use bacterial EMVs, as these are safe, cheap to produce, and provide high levels of protection [9]. Indeed, several EMV vaccines against serogroup B* Neisseria meningitides* (MenB) have been licensed for human use in Norway, Cuba, Chile, and New Zealand [10–13]. Here, we showed that* S. pneumoniae* EMVs are both effective against* S. pneumoniae* infection and safe in an animal model (Figures 2 and 3). This supports the notion that bacterial EMVs have great potential as next-generation vaccines that protect humans from bacterial infections.

We identified 61 putative antigenic proteins (extracellular, membrane, and cell wall proteins) that may be recognized by the host immune system and induce adaptive immunity. Previous reports identified the immunogenic proteins in exoproteomes of* S. pneumoniae* [14, 15]. We also found some of these immunogenic proteins in* S. pneumoniae* EMVs, namely, MalX, AliA, Ami, PspA, Eno, ABC-SBP, Sphra, and ZamB (Supplementary Table S1). These results suggest that the proteins in the EMVs may be immunogenic antigens that induce the production of specific antibodies by the host. Previously, existence of EMVs from other Gram-positive bacteria and their protein composition was reported. 90 proteins from* Staphylococcus aureus* EMVs [16], 104 proteins from* Bacillus anthracis* EMVs [17], and 426 proteins were identified from* Clostridium perfringens* EMVs [18]. Among the immunogenic proteins identified in* S. pneumoniae*, only MalX, Eno, and Sphra were discovered in* C. perfringens*. Therefore, these results suggest that the other immunogenic proteins may be specific for* S. pneumonia *and each bacterium has different immunogenic protein sets. These proteins can be used to develop diagnostic kits and subunit vaccines for* S. pneumoniae*.

In this study, we isolated the EMVs from a nonpathogenic* S. pneumoniae* strain (BAA-255), which has no polysaccharide capsule. The main difference between pathogenic and nonpathogenic* S. pneumoniae* is the presence of a polysaccharide capsule in the former. This polysaccharide capsule is the most potent virulence factor [19]. Capsular polysaccharide is also used as pneumococcal vaccines. Our results showed that immunization with the EMVs from the nonpathogenic* S. pneumoniae* strain BAA-255 protected mice from infection with a pathogenic* S. pneumoniae* strain (KCCM-41569), especially when multiple vaccinations were given (Figure 3(b)). This suggests that EMV proteins are responsible for acquired immunity against bacterial infection. For immunization, we inoculated EMVs only. Therefore, less EMVs would be sufficient for immunization when they are inoculated with adjuvants.

*S. pneumoniae* BAA-255 is a nonpathogenic noncapsulated strain and we found that its EMVs had no cytotoxic effect on A549 lung cancer cells, even at high concentrations (200 *μ*g) (Figure 2). In fact, the EMVs from nonpathogenic* S. pneumoniae* BAA-255 was even safer than the EMVs from the environmental soil bacterium* Pseudomonas putida *KT2440: a previous study showed that 25 *μ*g of* P. putida* KT2440 EMVs induced early apoptosis in A549 cells [7]. The difference between* S. pneumoniae *and* P. putida* EMVs with respect to cytotoxicity may be due to the fact that* P. putida* is Gram-negative: the EMVs from Gram-negative bacteria often express endotoxic lipopolysaccharide (LPS) on their outer membrane (these EMVs are commonly known as outer membrane vesicles) [20]. Indeed, EMVs from many Gram-negative bacteria are cytotoxic [21]. Since* S. pneumoniae* BAA-255 is Gram-positive, its EMVs do not carry LPS. Thus, the EMVs of Gram-positive bacteria may be safer and more suitable for vaccines than EMVs derived from Gram-negative bacteria.

## 5. Conclusions

The present study aimed to examine the potential of bacterial EMVs as vaccines. The results showed that EMVs isolated from* S. pneumoniae* BAA-255 protected mice with no notable side effects. In addition, we identified the immunogenic proteins that are expressed on* S. pneumoniae* BAA-255 EMVs. Since EMVs are more immunogenic than an equivalent amount of bacterial cell extract [15], these findings suggest that EMVs are highly promising as potential vaccine antigens.

## Supplementary Material

Supplementary material includes a figure of cytotoxicity assay of pathogenic S. Pneumoniae KCCM-41569 and a table of list of total proteins identified in S. Pneumoniae BAA-255 EMVs.
