# Supplementary material for: Potential Usefulness of Streptococcus pneumoniae Extracellular Membrane Vesicles as Antibacterial Vaccines
Source: J Immunol Res. 2017 Jan 22;2017:7931982. doi: 10.1155/2017/7931982 (PMC5292160; doi:10.1155/2017/7931982)
Supplement: Supplementary file 1 — Supplementary material includes a figure of cytotoxicity assay of pathogenic S. Pneumoniae KCCM-41569 and a table of list of total proteins identified in S. Pneumoniae BAA-255 EMVs. [file 7931982.f1.pdf]

Figure S1

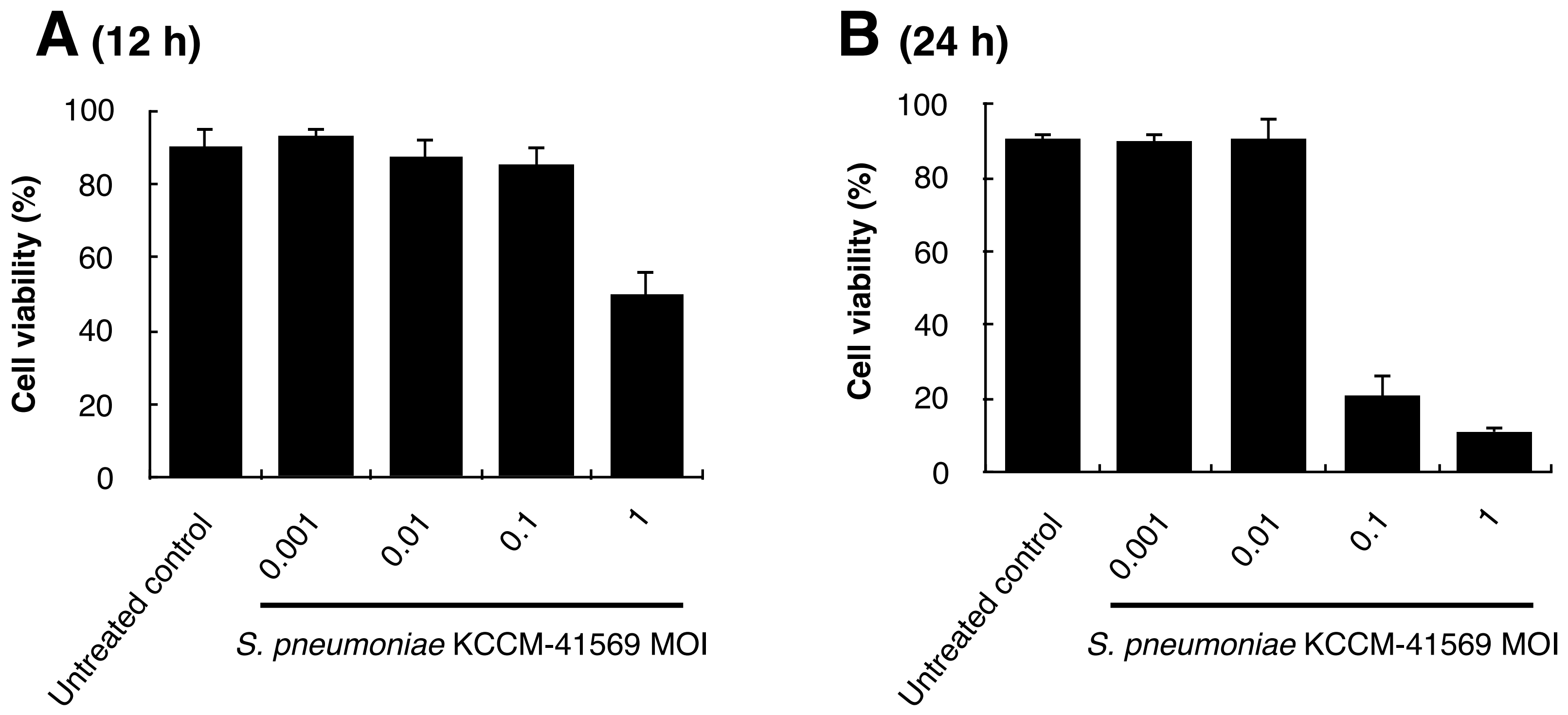

Figure 2: Cytotoxicity of pathogenic *S. pneumoniae* KCCM-41569. A549 cells were treated with various concentrations of intact *S. pneumoniae* KCCM-41569 and cell viability were analyzed after 12 h (A) and 24 h (B).

Supplementary Table S1. Total protein identification of S. pneumoniae BAA-255 EMVs

Accesions	sp	Description	Mw	pI	Length	Gene	mol%	STD
gi|15902791	spr0747	hypothetical protein spr0747	39481	5.46	374	-	9.388469972	0.1560434
gi|15903537	spr1494	ABC transporter substrate-binding protein - manganese transport.	34573	5.3	309	psaA	8.669140754	2.1939489
gi|15902928	spr0884	foldase protein PrsA	34419	5.04	313	prsA	6.587788711	2.3312583
gi|15903959	spr1918	maltose/maltodextrin ABC transporter, maltose/maltodextrin-binding protein	45339	5.1	423	malX	6.12436041	1.543136
gi|15902703	spr0659	branched chain amino acid ABC transporter amino acid-binding protein	40389	5.29	386	livJ	4.398227517	1.4002809
gi|15902190	spr0146	ABC transporter substrate-binding protein - amino acid transport	30609	5.14	276	ABC-SBP	3.452578457	0.222098
gi|15902371	spr0327	ABC transporter substrate-binding protein - oligopeptide transport	72958	5	660	aliA	3.241318074	0.0655517
gi|15902978	spr0934	iron-compound ABC transporter, iron compound-binding protein	37483	5.38	341	ABC-SBP	3.174657417	0.7049358
gi|15903294	spr1251	amino acid ABC transporter amino acid-binding protein	29246	4.96	271	glnH	2.973412961	0.3965419
gi|15902975	spr0931	hypothetical protein spr0931	35377	5.46	324	-	2.493663502	0.4747721
gi|15902065	spr0021	adenylosuccinate synthetase	47541	5.49	428	purA	2.279355635	0.8030742
gi|15903749	spr1707	ABC transporter substrate-binding protein - oligopeptide transport	72460	4.95	659	amiA	2.218015688	0.1863712
gi|15902598	spr0554	hypothetical protein spr0554	26522	5.13	238	-	2.009531371	0.6770069
gi|15902723	spr0679	peptidyl-prolyl cis-trans isomerase, cyclophilin-type	28991	5.47	267	ppiA	1.93556356	0.4604236
gi|15902165	spr0121	surface protein pspA precursor	68563	5.12	619	pspA	1.518440879	0.1636823
gi|15902191	spr0147	lipoprotein	31128	5.46	284	ABC-SBP	1.507207113	0.1033003
gi|15903396	spr1353	amino acid ABC transporter amino acid-binding protein	31000	4.83	278	glnH	1.485501739	0.2191758
gi|15902732	spr0688	hypothetical protein spr0688	12929	6.97	112	-	1.409026917	0.6226718
gi|15903080	spr1036	phosphopyruvate hydratase	47074	4.7	434	eno	1.340063239	0.5065567
gi|15903882	spr1840	preprotein translocase subunit YajC	11040	5.39	99	yajC	1.289986435	0.3375
gi|15904016	spr1975	zinc ABC transporter zinc-binding protein	56237	5.05	501	adcA	1.142366497	0.2493074
gi|15903824	spr1782	hypothetical protein spr1782	37514	5.26	345	-	1.069879734	0.118677
gi|15902408	spr0364	signal peptidase I	23483	5.84	204	spi	1.04977368	0.1415889
gi|15903425	spr1382	ABC transporter substrate-binding protein - oligopeptide transport	72517	5.24	652	aliB	0.990323865	0.0166318
gi|15903953	spr1912	hypothetical protein spr1912	11376	6.6	103	-	0.975651634	0.5880118
gi|15902247	spr0203	30S ribosomal protein S8	14745	9.58	132	rpsH	0.883961593	0.2457316
gi|15903300	spr1257	phosphate ABC transporter phosphate-binding protein	31182	4.77	292	pstS	0.882555802	0.2299703
gi|15902628	spr0584	glucokinase	34164	4.95	325	glcK	0.880138976	0.0923384
gi|15903019	spr0975	hypothetical protein spr0975	36417	7.74	344	ABC-SBP	0.876536294	0.1481765
gi|15903570	spr1527	sugar ABC transporter, sugar-binding protein	48273	5.31	442	ABC-SBP	0.850488545	0.5940455
gi|15904044	spr2003	ABC transporter substrate-binding protein	37753	5.41	335	-	0.835934711	0.0168729
gi|15902249	spr0205	50S ribosomal protein L18	12859	10.4	118	rplR	0.770796941	0.0155581
gi|15903143	spr1100	L-lactate dehydrogenase	35333	5.09	328	ldh	0.756982415	0.4160856
gi|15903295	spr1252	phosphate transporter PhoU	25013	4.73	217	phoU	0.756373122	0.3022053
gi|15902630	spr0586	hypothetical protein spr0586	6002	10.5	56	-	0.736634661	0.2047763
gi|15902243	spr0199	50S ribosomal protein L14	12998	10.27	122	rplN	0.732580582	0.100082
gi|15902250	spr0206	30S ribosomal protein S5	17046	9.52	164	rpsE	0.725343052	0.1051522
gi|15903413	spr1370	hypothetical protein spr1370	60776	5.07	551	-	0.665799545	0.0788021
gi|15903237	spr1194	ABC transporter substrate-binding protein - oligopeptide transport	61169	5.86	542	appA	0.651162521	0.1170323
gi|15902316	spr0272	30S ribosomal protein S9	14226	10.86	130	rpsI	0.646993075	0.2897589
gi|15904003	spr1962	hypothetical protein spr1962	33573	5.62	299	-	0.542745657	0.08502
gi|15902373	spr0329	penicillin-binding protein 1A	79652	5.41	719	pbpA	0.531742189	0.2209267
gi|15902425	spr0381	3-ketoacyl-(acyl-carrier-protein) reductase	25753	5.49	243	fabG	0.521510974	0.0544111
gi|15902422	spr0378	acyl carrier protein	8263	3.64	74	acpP	0.521102157	0.0105182
gi|15903387	spr1344	glycerol uptake facilitator protein, putative	30652	9.02	289	glpF	0.494321395	0.1211294
gi|15902737	spr0693	hypothetical protein spr0693	41839	5.12	399	-	0.493071081	0.2571935
gi|15903500	spr1457	hypothetical protein spr1457	16787	5.03	158	-	0.472079771	0.2699736
gi|15903966	spr1925	hypothetical protein spr1925	8391	10.07	76	-	0.46790911	0.0732834
gi|15902921	spr0877	preprotein translocase subunit SecG	8545	9.69	77	secG	0.449980038	0.180402
gi|15903867	spr1825	glyceraldehyde-3-phosphate dehydrogenase	38739	5.78	359	gapA	0.429200782	0.1245704
gi|15903480	spr1437	sugar ABC transporter, ATP-binding protein	41808	5.83	376	msmK	0.417769088	0.1305656
gi|15903206	spr1163	cytoplasmic membrane protein	20621	5.66	186	lemA	0.416313067	0.0304479
gi|15902485	spr0441	phosphoglycerate kinase	41913	4.92	398	pgk	0.382818996	0.1488013
gi|15903687	spr1645	ABC transporter substrate-binding protein	39178	5.61	355	ABC-SBP	0.36699224	0.028291
gi|15903058	spr1014	50S ribosomal protein L27	9528	10.44	89	rpmA	0.347401438	0.0070121
gi|15903542	spr1499	phosphoglyceromutase	26035	5.14	230	gpmA	0.339174688	0.1370685
gi|15902526	spr0482	ribosome-binding factor A	14456	9.36	125	rbfA	0.333347465	0.1051892
gi|15902055	spr0011	hypoxanthine-guanine phosphoribosyltransferase	20199	5.26	180	hgt	0.328633057	0.0445711
gi|15902501	spr0457	hypothetical protein spr0457	10882	5.09	95	-	0.328479474	0.0975799
gi|15902293	spr0249	30S ribosomal protein S7	17745	10.29	156	rpsG	0.327607999	0.1515104
gi|15903739	spr1697	hypothetical protein spr1697	11402	10.37	100	-	0.323278433	0.1128454
gi|15903163	spr1120	amino acid ABC transporter permease/amino acid-binding protein	78318	8.39	721	glnP	0.323278433	0.1128454
gi|15902238	spr0194	50S ribosomal protein L22	12193	10.76	114	rplV	0.322142102	0.0231057
gi|15902245	spr0201	50S ribosomal protein L5	19762	9.04	180	rplE	0.314832553	0.0063547
gi|15902218	spr0174	hypothetical protein spr0174	21027	5.83	189	-	0.301718339	0.0173966
gi|15902686	spr0642	pyruvate oxidase	65214	5.03	591	spxB	0.293119964	0.0059165
gi|15903548	spr1505	cell division protein DivIVA	30724	4.7	266	divIVA	0.293119964	0.0059165
gi|15903729	spr1687	iron-compound ABC transporter, iron-compound-binding protein	34841	5.55	321	fatB	0.292846458	0.1394699
gi|15903155	spr1112	hypothetical protein spr1112	20260	10.38	187	-	0.287022781	0.0690185
gi|15903406	spr1363	F0F1 ATP synthase subunit delta	20528	7.77	178	atpH	0.276866119	0.1919714
gi|15902820	spr0776	D-alanyl-D-alanine carboxypeptidase	45192	4.87	413	dacA	0.272867144	0.0826595

gi|15903468	spr1425	endoribonuclease L-PSP	13723	4.67	126	aldR	0.271407374	0.0054782
gi|15903874	spr1832	hypothetical protein spr1832	27975	7.74	242	-	0.271032486	0.1530246
gi|15902959	spr0915	large conductance mechanosensitive channel protein MscL	13608	9.78	125	mscL	0.264673928	0.1208711
gi|15903183	spr1140	hypothetical protein spr1140	13702	9.75	119	-	0.263399752	0.1463909
gi|15902304	spr0260	PTS system, mannose-specific IIC component	27213	4.84	267	manM	0.260551079	0.0052591
gi|15902639	spr0595	hypothetical protein spr0595	14568	10.05	126	-	0.24612923	0.0972089
gi|15902659	spr0615	hypothetical protein spr0615	14431	8.76	134	-	0.238838489	0.0048208
gi|15904064	spr2023	rod shape-determining protein MreC	29721	5.87	272	mreC	0.235053807	0.0861335
gi|15903310	spr1267	hypothetical protein spr1267	14304	4.43	129	-	0.21968881	0.0646329
gi|15902950	spr0906	adhesion lipoprotein	34678	5.27	311	lmb	0.210457999	0.0498477
gi|15903482	spr1439	transcriptional repressor CodY	29738	5.55	262	codY	0.207146119	0.0475886
gi|15903100	spr1056	hypothetical protein spr1056	25660	6.34	215	-	0.206269604	0.0041634
gi|15902240	spr0196	50S ribosomal protein L16	15426	10.66	137	rplP	0.204740744	0.0135784
gi|15903403	spr1360	F0F1 ATP synthase subunit beta	50872	4.85	468	atpD	0.198087557	0.0424541
gi|15903520	spr1477	Rrf2 family protein	15814	5.54	145	-	0.195413309	0.0039443
gi|15904086	spr2045	serine protease	42262	6.15	397	sphtra	0.18567515	0.0580292
gi|15902303	spr0259	phosphotransferase system, mannose-specific EIID	33764	8.79	303	manN	0.17625859	0.0191594
gi|15902315	spr0271	50S ribosomal protein L13	16133	9.95	148	rplM	0.169477779	0.0631061
gi|15903602	spr1559	ABC transporter ATP-binding protein - unknown substrate	26664	5.71	240	ABC-NBD	0.164591325	0.0590399
gi|15903943	spr1902	NAD(P)H-dependent glycerol-3-phosphate dehydrogenase	36754	5.19	338	gpsA	0.156920579	0.0461663
gi|15903255	spr1212	50S ribosomal protein L10	17469	5.08	166	rplJ	0.15220617	0.1077875
gi|15903296	spr1253	phosphate ABC transporter ATP-binding protein	27936	5.36	252	pstB	0.151988129	0.0030678
gi|15903443	spr1400	hypothetical protein spr1400	18311	6.6	162	-	0.14007094	0.0445949
gi|15903199	spr1156	pyrimidine regulatory protein PyrR	19597	5.35	173	pyrR	0.126415435	0.0352907
gi|15903720	spr1678	choline transporter	27082	4.97	242	proV	0.123783434	0.0386861
gi|15902127	spr0083	ABC transporter substrate-binding protein	57190	5.94	514	ABC-SBP	0.123783434	0.0386861
gi|15903473	spr1430	non-heme iron-containing ferritin	20003	4.67	178	dpr	0.119419244	0.0024104
gi|15903376	spr1333	peptidoglycan GlcNAc deacetylase	52642	5.45	463	pgdA	0.109844405	0.0323164
gi|15902671	spr0627	lactate oxidase	41463	5.67	378	lctO	0.108310504	0.0338503
gi|15902824	spr0780	PTS system, fructose specific IIABC components	66892	5.31	650	fruA	0.097706655	0.0019722
gi|15902625	spr0581	Zinc metalloprotease	210928	5.3	1876	zmpB	0.08685036	0.001753
gi|15902948	spr0904	hypothetical protein spr0904	21348	7.59	191	-	0.062651854	0.0286823
gi|15902200	spr0156	hypothetical protein spr0156	22779	6.9	200	-	0.059380929	0.0150792
